# New Appliances and Things Medical

**Published:** 1896-07-25

**Authors:** 


### NEW APPLIANCES AND THINGS MEDICAL.

[We shall be glad to receive, at our Office, 428, Strand, London, W.C., from the manufacturers, specimens of all new preparations and appliances, which may be brought out from time to time.]

### OLD SCOTCH WHISKY (BIG BEN).

(J. L. Denman and Co., Glasgow and London.)

A sample of this whisky has been forwarded to us, and we find it of excellent quality, well matured, and of agreeable blend. It is a spirit which may be safely used for invalid purposes, and for those in health will be found to be a stimulant of great excellence.

### PETROLEUM JELLY (SNOWDRIFT BRAND).

(Messrs. Snowden, Sons, and Co., Millwall, E.)

We have received from Messrs. Snowden, Sons, and Co. a sample of their Snowdrift Brand Petroleum Jelly, a special new make of extra quality. We have had occasion before to draw attention to the fact that, after careful analysis, this firm's petroleum jellies are recognised as among the very best which it is possible to obtain. After carefully examining this Snowdrift brand, we are of opinion that its tough or stringy nature is a great improvement. It is again with pleasure we find that a petroleum jelly of such a superior quality (which appears to be nothing more or less than a pure vaselene) can be placed on the market by an English firm. We learn that already the demand for the Snowdrift brand is very great. It is cheaper than ordinary vaseline, and we confidently recommend it to the medical profession.

### TRITICINE—A FOOD FOR INFANTS AND INVALIDS.

(Goodall and Sons, Castleford.)

When made according to the directions supplied Triticine constitutes a most palatable food; it can be readily absorbed from the alimentary tract, and little or no *débris* is left behind to irritate a delicate mucous membrane, or set up those fermentative processes which are the chief cause of infant diarrhœa. On examining the analysis which has been made by Mr. James Baynes, public analyst for the East Riding of Yorkshire, it is obvious that it contains a rich proportion of readily assimilable carbohydrates and albuminoids, and that there is a sufficiency of phosphates, while there is a commendable absence of cellulose, or the less easily digested part of the cereal. The proportion of fat, however, 1·12, renders it imperative that Triticine must be made with a proportion of milk to constitute a complete food.

